# Photosynthetic and ascorbate-glutathione metabolism in the flag leaves as compared to spikes under drought stress of winter wheat (*Triticum aestivum* L.)

**DOI:** 10.1371/journal.pone.0194625

**Published:** 2018-03-22

**Authors:** Lili Lou, Xiaorui Li, Junxiu Chen, Yue Li, Yan Tang, Jinyin Lv

**Affiliations:** College of Life Sciences, Northwest A&F University, Yangling, Shaanxi, China; Universidade de Lisboa Instituto Superior de Agronomia, PORTUGAL

## Abstract

Ascorbate-glutathione (ASA-GSH) cycle is a major pathway of H_2_O_2_ scavenging and an effective mechanism of detoxification in plants. The differences in photosynthesis, chlorophyll content (Chl), relative water content (RWC), antioxidants and antioxidative enzyme activities involved in ASA-GSH metabolism were measured between the flag leaves and spike bracts (glumes and lemmas) during grain filling under drought stress. The expression of *APX1*, *GRC1*, *DHAR*, *MDHAR*, *GPX1*, and *GS3* in ASA-GSH cycle was also measured. Compared with the flag leaves, the spike bracts exhibited stable net photosynthetic rate (*P*_*N*_) and chlorophyll content (Chl), a lower accumulation of reactive oxygen species (ROS), and more enhanced percentages of antioxidant enzyme activities and key enzymes gene transcription levels involved in ASA-GSH metabolism during the grain-filling stage under drought conditions. This could be the reasonable explanation for the more stable photosynthetic capacity in spikes, and the glumes and lemmas senesced later than the flag leaves at the late grain-filling stage. Also, the function of ASA-GSH cycle could not be ignored in alleviating oxidative damage by scavenging more excess ROS in spikes under drought stress.

## Introduction

Drought has become the most important threat limiting plant growth, development, and crop production [1–4]. In recent years, crop production is challenged with frequently occurring drought periods around the world’s arid regions. It is reported that cereal production causing by the reduction of available water was significantly decreased by roughly 10% on average [5]. Wheat (*Triticum aestivum* L.) is one of the largest cultivated cereals in the world [6], and drought is a major factor resulting in the decrease of yields and quality of wheat [7], especially post-anthesis drought [8]. Northern China is one of the main regions of wheat production, accounting for roughly 85% of summer grain yields in China [9]. Unfortunately, due to the monsoon climate, rainy season in this region is inconsistent with the growth period of wheat. As a result, water deficit becomes a common occurrence at the time of grain-filling, which causes a severe reduction in production [10, 11]. Photosynthesis is the key process of plant metabolism which is strongly influenced by environmental conditions [12, 13]. In the last decades, a number of studies evaluated the contribution of ear photosynthesis to grain-filling in C_3_ cereals, and revealed that ear photosynthesis contributes largely to grain yields [14–21], especially under water deficit conditions and that the ear may become the main photosynthetic organ maximizing carbon assimilation [22–25]. In fact, it has been suggested that “*high-spike photosynthesis*” is an important trait for drought tolerance in wheat [26] and “*C*_*4*_ *photosynthetic pathway*” exists in the developing wheat grain [27]. However, Busch and Farquhar [28] think that the evidence is poor for C_4_ photosynthesis in wheat grain. Therefore, more experimental evidences are needed for this controversial issue. Although the C_4_ metabolism in the ear is not completely understood, it seems to be associated with many features of spikes for adaptation to drought stress such as high relative water content and water use efficiency [29], xeromorphic anatomy [15], better osmotic adjustment [30], and delayed senescence [31] in glumes, lemmas and awns. Moreover, ear bracts were able to re-fix the CO_2_ respired by growing grains [19]. However, the molecular mechanism of photosynthesis in spikes under drought stress during grain-filling is not completely understood.

It is widely accepted that reactive oxygen species (ROS) has a dual effect which is based on their overall cellular amount in plants [32, 33]. If kept in a low level, they can function as signaling molecules to transmit information from metabolism to trigger appropriate cellular defense/acclimation responses to developmental and environmental changes [34, 35]. However, they were originally thought to be formed in different compartments of cells as “an unavoidable toxic byproducts of aerobic metabolism” which was amplified under stress conditions [36–38], and it will lead to an imbalance of the cellular redox potential and ultimately to programmed cell death [39]. As we all know, plants will trigger the production of ROS in response to drought stress [40]. The quantity of ROS in cells is influenced by the antioxidative system and a number of metabolic pathways [41].

The ascorbate-glutathione (ASA-GSH) cycle, one of the most important components of the antioxidative system, is a major pathway of H_2_O_2_ scavenging and an effective mechanism of detoxification in plant cells by utilizing ASA and GSH, and their associated enzymes. It is composed of monodehydroascorbate reductase (MDHAR), dehydroascorbate reductase (DHAR), ascorbate peroxidase (APX), glutathione reductase (GR) and glutathione peroxidase (GPX) [42–44]. Compounds such as ASA and GSH are simple antioxidants that can directly quench ROS functioning as cofactors of the antioxidative enzymes, especially they have reduction and oxidation states [45]. Most importantly, antioxidants provide the necessary information about cellular redox state, and they affect gene transcription levels related with stress responses to maximize defense. In fact, the protective role of ASA-GSH cycle in alleviating oxidative damage caused by drought was suggested to be closely associated with its participation in regulating the gene expression of key enzymes including *APX1*, *GRC1*, *DHAR*, *MDHAR*, *GPX1*, and *GS3* (glutathione synthetase) involved in ASA-GSH cycle, which are responsible for the regulation of oxidative status [46–48]. Responses of related genes in spike bracts to water stress have been largely overlooked even though the ear photosynthesis is the main provider of carbon to the developing seed with the crop reduction under drought stress. To achieve a more comprehensive understanding of the mechanism of drought tolerance between the flag leaves and spike bracts during grain-filling, we combined photosynthetic parameters, key antioxidant enzymes and antioxidants involved in ASA-GSH cycle, and key enzymes gene expression analysis to help us understand much better the differences in physiological and metabolic responses between the flag leaves and spike organs during drought, which can investigate the drought tolerance mechanism in wheat.

## Materials and methods

### Plant material and growth condition

A pot culture experiment was performed from October 2015 to June 2016 in the Northwest A&F University, Yangling (N 34°22′, E 108°26′, 526 m elevation), Shaanxi Province, China. The common winter wheat cultivar Pubing 143 was used in present study.

Surface soil (0–20 cm) was collected from the local agricultural farm (Earth-cumuli-Orthic Anthrosols). The field water capacity was 29.2%. It was passed through a 0.5 cm sieve. Then 8.0 kg of soil was filled with each plastic pot (24 cm in upper diameter, 17 cm in bottom diameter, and 24 cm in height) after artificially mixed with urea (0.347 g kg^–1^ soil) and KH_2_PO_4_ (0.2 g kg^–1^ soil). The soil subsamples were fully watered and then left for equilibration for approximately 20 days. In each pot, 20 seeds were sown directly into the soil, and 12 main stems were retained per pot after removing tillers at the jointing stage. Water controlling was initiated in the late elongation stage (April 12, 2016) by weighing method. The soil water content of control treatment (Well-watered, WW) was kept at 20.44–21.9% (70–75% of field water capacity), and drought treatment (Water-stressed, WS) was kept at 10.22–11.68% (35–40% of field water capacity). The pots were weighted every other day, and water was supplemented until the soil moisture content reached the standard. A total of 30 replicate pots per treatment were prepared. The flag leaves and spike parts (glumes and lemmas) of wheat (S1 Fig) were harvested at 0, 5, 10, 15, 20, and 25 days after anthesis (DAA).

### Photosynthetic parameters of the flag leaves and spikes

The net photosynthetic rate (*P*_N_), transpiration rate (*E*), and stomatal conductance (*g*_s_) of the flag leaves were directly measured by a portable gas-exchange photosynthesis system (*LI-6400 XT*, *LiCor*, USA) in accordance with the method described by Jia [24]. Photosynthetic parameters of spikes were measured using a special cylindrical chamber that was made of glasses and connected to the portable photosynthetic system [49]. Photosynthesis measurements were performed at 9:00 a.m. to 11:00 a.m. with a saturating photosynthetic photon flux density (PPFD) of 1,000 μmol (photon) m^–2^ s^–1^ at 0, 5, 10, 15, 20, and 25 DAA with five replicates for each treatment. The length and width of leaf area was measured with a ruler. The calculation of ear surface area was according to the formula of the fringe area [50–52].

$$\begin{array}{l} \text{Whole}\;\text{ear}\;\text{area}=\text{ear}\;\text{length}\times \text{ear}\;\text{width}\times 3.8\;\left(\text{glumes}\;\text{surface}\;\text{area}\right)\\ +\text{total}\;\text{awn}\;\text{length}\;\text{of}\;\text{top}\;\text{third}\;\text{spikelet}\times \text{fruit}\;\text{spikelet}\;\text{number}\\ \times 0.1\;\left(\text{awn}\;\text{surface}\;\text{area}\right) \end{array}$$

### Chlorophyll content (Chl) and relative water content (RWC)

The Chl assay was a modification of the method of Evans [53]. The Chl was extracted from fresh samples in cold 80% acetone at 4°C for 24 h. The absorbance of the extracts was determined by a UV-Vis spectrophotometer (Shimadzu, Kyoto, Japan) at 645 and 663 nm. Analyses were carried out in quadruplicates. The total Chl content was calculated as x mg g^–1^(FM) = 20.21 OD_645_ + 8.02 OD_663_.

Relative water content (RWC) was determined according to Smart [54]. The fresh mass (FM) was weighted immediately after the sample collection, followed by flotation on distilled water for 12 h in darkness at a low temperature (4°C), and then the turgid mass (TM) was recorded. Finally, the sample was dried at about 80°C to a constant weight and the total dry mass (DM) was recorded. RWC was calculated by:

RWC[%]=(FM–DM)/(TM–DM)×100%.

### Determination of hydrogen peroxide (H_2_O_2_) and malondialdehyde (MDA) contents

The H_2_O_2_ content was estimated spectrophotometrically according to Alexieva [55]. Hydro-gen peroxide was measured spectrophotometrically after a reaction with KI. The reaction mixture consisted of 0.5 mL 0.1% trichloroacetic acid (TCA) leaf extract supernatant, 0.5 mL of 100 mM K-phosphate buffer and 2 mL reagent (1M KI w/v in fresh double-distilled water H_2_O). The blank probe consisted of 0.1% TCA in the absence of leaf extract. The reaction was developed for 1 h in darkness and absorbance was measured at 390 nm. The amount of hydrogen per-oxide was calculated using a standard curve prepared with known concentrations of H_2_O_2_.

The MDA content was determined following the method described by Li [56]. A mixture of 1 mL of supernatant and 4 mL of reaction solution (TCA reactive substances (with 0.5% in 20% TCA)) was heated by incubating at 95 °C for 25min and immediately cooled in ice bath. The mixture was centrifuged at 12 000 g for 10min, and supernatant was used to determined the MDA content at 532 nm and 600 nm.

### Assays of total GSH, GSSG, total ascorbate and reduced ascorbate contents

Total GSH and GSSG contents were measured following the procedure described by Anderson [57]. Samples (0.2 g) were homogenized in 2 mL of 5% sulfosalicylic acid at 4 °C. The homogenate was centrifuged at 12,000 g for 10 min. Approximately 100 μL of the supernatant was added to 100 μL of 5% sulfosalicylic acid, and the mixture was neutralized by adding 48 μL of 1.84 M triethanolamine. About 50 μL of the sample was used to determine total GSH (GSH+GSSG). Another 50μL of the sample was pretreated with 50 μL of 2-vinylpyridine for 60 min at 25 °C to mask GSH by derivatization and allow the determination of GSSG alone. Both types of samples were added with 20 μL of 10 mM NADPH, 80 μL of 12.5 mM DTNB, and 706 μL of 50 mM phosphate buffer (pH 7.5) containing 2.5 mM EDTA. Approximately 20 μL of GR (50 U/mL) was then added, and changes in absorbance were monitored at 412 nm. The GSH content was estimated from the difference between total GSH (GSH+GSSG) and GSSG.

The method described by Masato [58] with some modifications was used to determine the total ascorbate and reduced ascorbate contents. Total AsA (AsA+DHA) was determined after DHA reduced to AsA with dithiothreitol (DTT), and the DHA content was estimated from the difference between the total AsA and reduced AsA. Samples (0.2 g) were extracted in ice-cold 5% (w/v) meta-phosphoric acid and centrifuged at 22,000 g for 15 min at 4 °C. For total AsA determination, the reaction mixture comprised 0.3 mL of the supernatant, 0.75 mL of 150 mM phosphate buffer (pH 7.4) containing 5 mM EDTA and 0.15 mL of 10 mM DTT. After incubation for 10 min at 25 °C, the solution was added with 0.15 mL of 0.5% N-ethylmaleimide to remove excess DTT. For AsA determination, a similar reaction mixture was used except that 0.3 mL of H_2_O was added rather than DTT and N-ethylmaleimide. Color was developed in both reaction mixtures after adding the following reagents: 0.6 mL of 10% TCA, 0.6 mL of 44% ortho-phosphoric acid, 0.6 mL of 4% α, α′-dipyridyl in 70% ethanol and 0.3% (w/v) FeCl_3_. After a vortex mixing, the mixture was incubated at 40 °C for 40 min and the absorbance was recorded at 525 nm.

### Determination of enzyme activities

To measure the GPX, APX, GR, MDHAR, DHAR enzymes activities, fresh materials were ground to a fine powder in a mortar with liquid nitrogen, then homogenized in 1 mL reaction contained 50 mM KH_2_PO_4_, 0.1 mM EDTA, and 0.3% (w/v) Triton X-100. For GR (EC 1.6.4.2), the Triton X-100 was omitted. Total protein was determined using bovine serum albumin as the standard for preparation of calibration curve following the method of Lowry and Rosebrough [59]. The APX (EC 1.11.1.11) activity measurement was performed following the method of Benabdellah [60]. The enzyme extract was added to the reaction mixture, which contained 50 mM Tris-HCl (pH 7.4), 0.5 mM EDTA, 0.25 mM NADPH, 2 mM H_2_O_2_, 1.0 mM NaN_3_, 2.25 mM GSH, and in a final volume of 1 mL. Reactions were initiated by adding 1.0 unit GR. The activity of GPX (EC 1.11.1.9) was determined by the decrease in absorbance at 340 nm. The APX activity was determined spectrophotometrically at 290 nm based on the method of Nakano and Asada. [61]. The GR activity was assayed by following the decrease in absorbance at 340 nm according to the method of Grace and Logan [62]. The activity of DHAR (EC 1.8.5.1) was assessed by record an increase in the absorbance level at 265 nm following the method of Pinto [63]. The MDHAR (EC 1.6.5.4) activity was assayed following the method of Pinto [63] with slight modifications. The reaction mixture(1 mL) contained 50 mM sodium phosphate buffer (pH 7.0), 0.25 U AAO, 2 mM ASA, 2 mM NADPH, and enzyme extract. One unit of MDHAR activity was defined as a 0.01 increase in absorbance at 290 nm for 1 min.

### RNA extraction and quantitative real-time polymerase chain reaction

Total RNA was isolated from plants using the TRIzol reagent (Invitrogen) based on the manufacturer’s instructions. The concentration and purity of RNA was determined by using an Epoch UV-Vis microplate spectrophotometer (BioTek). The first-strand cDNA was synthesized using reverse transcriptase system (DRR037A; Takara, Dalian, China). All primers used for qRT-PCR are listed in Table 1. For real-time PCR, each reaction contained 2.0 μL of cDNA, 10 μL of SYBR Premix Ex Taq TM II, gene-specific primers 1.6 μL, and distilled deionized water up to a final volume of 20 μL. The PCR parameters were 95°C for 30 s; followed by 40 cycles of 95°C for 5 s, 60°C for 30 s. The melting curves were performed for the amplification of 95°C for 10 s, 65°C for 5 s, 95°C for 5 s. The wheat tubulin gene was used as a reference gene [64], and the reactions were performed using the Thermal Cycler Dice Real Time System III (Takarabio Inc., Otsu, Shiga, Japan). The baseline data were gathered between 18 and 32 cycles. All reactions were run in triplicate. The quantification of gene expression levels was caculated as 2^−ΔΔCT^ relative to the control.

**Table 1:** DNA sequences of PCR primers were used for QRT-PCR determination of ASA-GSH biosynthesis-related genes in wheat.

Genes	Accession no.	Primer pairs
*APX1*	AY513263.1	F: AAAGCGAAGCATCCAAAG
		R: CAGAGGGTCACGAGTCCA
*GRC1*	AY364467	F: ATGAATACTCCCGTACATCAGT
		R: TTTGTTACATCACCCACAGC
*DHAR*	AY074784	F: GTGCCTGTGTATAACGGTG
		R: ACAAGTGATGGAGTTGGGT
*MDHAR*	AK371371	F: AGAAGTTTACGCCCTTCGGC
		R: TTGGAATGTCATCGCCATC
*GS3*	AJ579382	F: ATCGCCAAGCTCCGTCAATG
		R: ACAAGTCAGGGTTTTCAATCG
*GPX1*	AF475124	F: GGAAAGTCCTGCTTATTGT
		R: CTTCTCATCGCTATCTGGT
*Tubulin*	U76558.1	F: TTCTCCCGCATCGACCACAAGTT
		R: TCCAGGGCAGCAAGATCCTCACG

### Statistical analysis

Data were performed with analysis of variance (*ANOVA*). *Duncan*’s multiple range analysis at *P* < 0.05 was used to detect the significant differences using *SPSS* statistical software (SPSS 22.0 Inc., Chicago, IL, USA). Error bars represent standard deviation (SD).

## Results

### Photosynthetic parameters of the flag leaves and spikes

**Fig 1:**
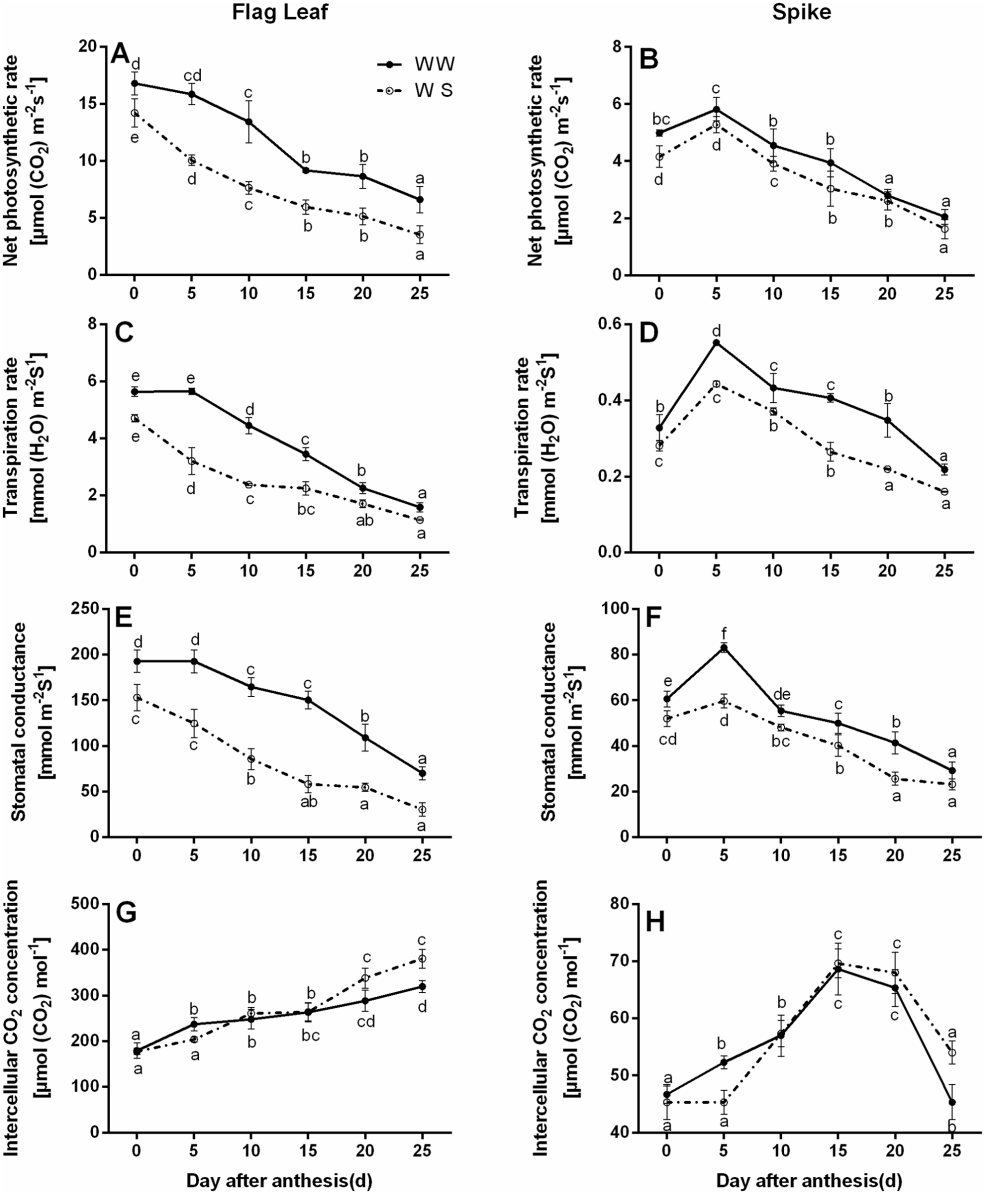
Photosynthesis parameters of the flag leaves (A, C, and E) and spikes (B, D, and F) at the grain-filling stage under well-watered (WW) and water-stressed (WS) conditions. All data represent means ± standard deviations (SD) of five replicates. Values with different letters indicated the significant differences at *P < 0*.*05* level in each stress treatment through time according to Duncan’s multiple range test. *P*_N_ – net photosynthetic rate; *g*_s_ – stomatal conductance; *E* – transpiration rate; *C*_i_ – Intercellular CO_2_ concentration; DAA – days after anthesis.

The photosynthetic parameters both in the flag leaves and spikes are shown in Fig 1. Wheat in WW condition showed higher *P*_N_ than that of the WS condition both in the flag leaves and spikes. The *P*_N_ of the flag leaves declined significantly during the grain-filling stage and this decline was faster in WS compared to the WW. But, the *P*_N_ of spikes increased to a maximum at 5 DAA and decreased thereafter. At the prometaphase of the grain-filling stage (10 DAA), the *P*_N_ of spikes decreased by 14.1% under WS, whereas the flag leaves were reduced by 42.9% compared with WW condition. The *E* and *g*_s_ showed similar trends with the *P*_N_ both in the flag leaves and spikes. In contrast, *C*_i_ showed an increasing trend during the grain-filling period in the flag leaves, and the value of *C*_i_ reached a peak at 15 DAA and then decreased in spikes.

### Chlorophyll content (Chl) and relative water content (RWC)

**Fig 2:**
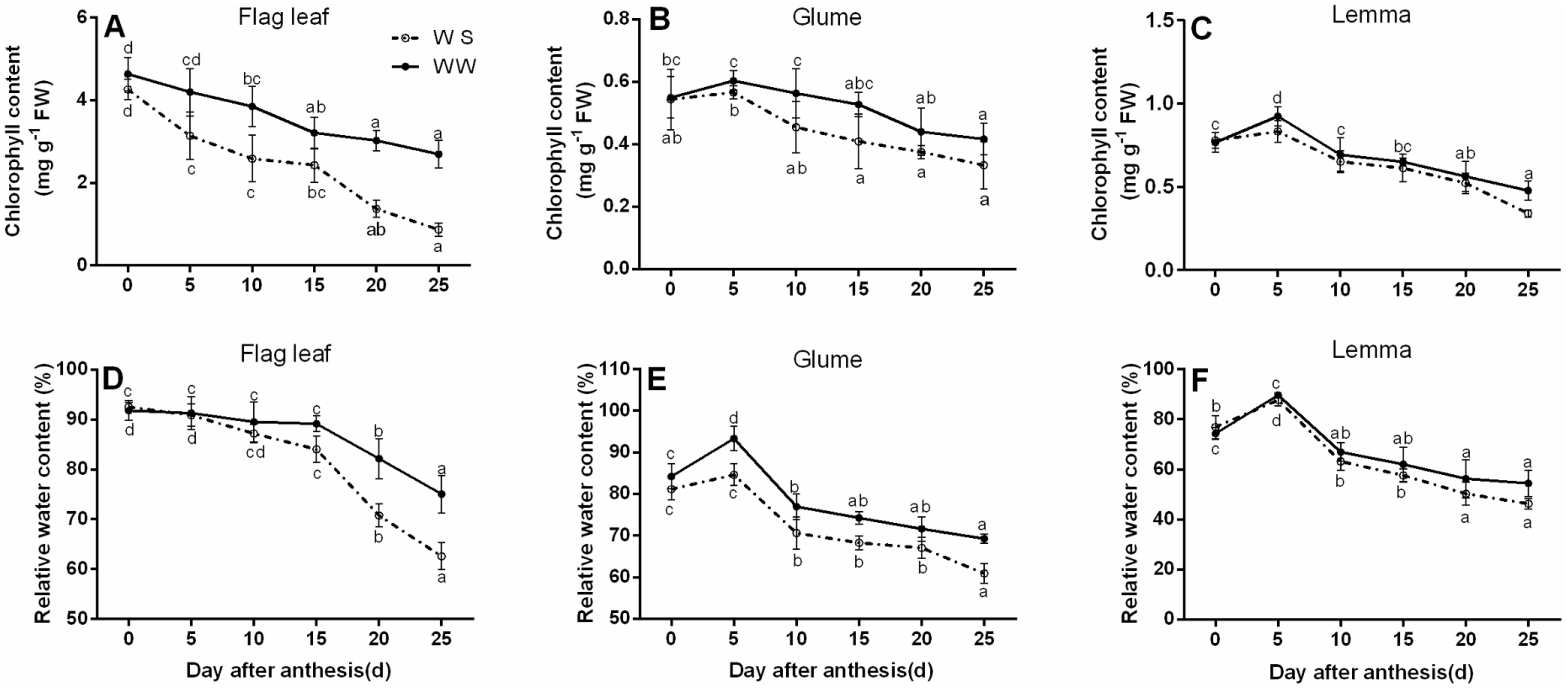
Chlorophyll content (Chl) and relative water content (RWC) of the flag leaves (A, D), glumes (B, E), and lemmas (C, F) at the grain-filling stage under well-watered (WW) and water-stressed (WS) conditions. All data represent means ± standard deviations (SD) of three replicates. Values with different letters indicated the significant differences at *P < 0*.*05* level in each stress treatment through time according to Duncan’s multiple range test.

The Chl content decreased with the onward grain-filling period both in the flag leaves and spikes, and it decreased much more quickly in the flag leaves than in non-leaf organs (glumes and lemmas). In later stage of grain-filling (20 DAA), compared with WW condition, the Chl content of glumes and lemmas decreased by 13.6% and 7.14% under WS, respectively. Whereas the Chl content of the flag leaves was reduced by 54.8% (Fig 2A–2C).

The RWC of the flag leaves was reduced slightly at 0–15 DAA under WS, and then fell by 18.2% at 25 DAA compared with 0 DAA. It dropped significantly by 17.2% compared with WW at 25 DAA (Fig 2D). At 5 DAA, the RWC of glumes and lemmas reached a maximum value and then declined (Fig 2E and 2F). In WS condition, the RWC of the flag leaves decreased from 92.5% to 62.6%, which was the largest loss of water compared with other two organs. All two spike organs showed evidence of water conservation ability, which is an indicator of ability to maintain cellular water during drought. There was no evidence of water conservation in the flag leaves.

### The contents of H_2_O_2_ and MDA

The content of MDA in the flag leaves markedly increased under WS condition from 5 DAA compared with the WW, with the highest increase observed at 25 DAA (Table 2). A similar trend was observed in glumes. In lemmas, the content of MDA did not vary between days in 0–20 DAA during the grain-filling period, but significantly increased at 25 DAA.

**Table 2:** Malondialdehyde (MDA) and hydrogen peroxide (H_2_O_2_) contents of the flag leaves, glumes, and lemmas at the grain-filling stage under well-watered (WW) and water-stressed (WS) conditions.

Parameters	Treatments	0 DAA	5 DAA	10 DAA	15 DAA	20 DAA	25 DAA
MDA content in flag leaf	WW	2.04±0.23^a^	1.72±0.32^a^	1.91±0.21^a^	2.06±0.50^a^	3.19±0.49^b^	6.72±0.65^b^
	WS	2.25±0.46^a^	3.14±0.30^ab^	3.94±0.12^b^	4.09±0.45^b^	5.49±0.30^c^	7.35±0.56^d^
MDA content in glume	WW	1.31±0.19^a^	2.14±0.23^b^	2.71±0.19^b^	3.54±0.31^c^	3.88±0.22^c^	5.71±0.42^d^
	WS	1.29±0.07^a^	2.34±0.47^b^	2.74±0.15^b^	3.32±0.25^c^	4.03±0.46^c^	6.63±0.33^d^
MDA content in lemma	WW	1.01±0.16^a^	2.18±0.16^b^	2.22±0.31^b^	2.57±0.10^b^	2.82±0.24^b^	4.18±0.48^c^
	WS	2.19±0.40^a^	2.46±0.49^a^	2.69±0.48^a^	2.98±0.39^a^	3.16±0.36^a^	6.88±0.29^b^
H_2_O_2_ content in flag leaf	WW	44.97±4.27^a^	48.24±2.46^a^	51.52±2.75^a^	61.27±2.46^b^	64.81±4.72^b^	73.96±6.66^c^
	WS	42.48±4.09^a^	54.10±3.58^b^	60.49±4.32^b^	55.08±3.97^b^	74.16±4.73^c^	85.60±4.74^d^
H_2_O_2_ content in glume	WW	5.04±1.61^a^	7.53±1.17^b^	8.35±0.68^b^	9.96±1.05^b^	9.98±1.40^b^	17.46±1.65^c^
	WS	6.78±1.27^a^	7.86±0.89^a^	8.61±1.47^a^	10.93±0.70^b^	12.65±1.22^b^	20.87±1.74^c^
H_2_O_2_ content in lemma	WW	7.82±1.23^a^	8.86±0.88^ab^	10.14±1.78^ab^	11.00±1.36^c^	13.76±1.47^d^	14.61±1.61^d^
	WS	6.37±1.12^a^	10.57±1.08^b^	12.89±1.26^bc^	14.14±0.96^c^	15.47±1.54^c^	21.74±0.81^d^

All data represent means ± standard deviations (SD) of five replicates. Values with different letters indicated the significant differences at *P < 0*.*05* level in each stress treatment through time according to Duncan’s multiple range test.

The H_2_O_2_ content in WW and WS treatments also increased in a time-dependent manner. However, the H_2_O_2_ content in the flag leaves of WS-treated were significantly higher than those of spike organs after five days of drought. These results indicated that the spike organs could control the accumulation of MDA and H_2_O_2_ contents better induced by drought.

### The redox state of ascorbate and glutathione

**Fig 3:**
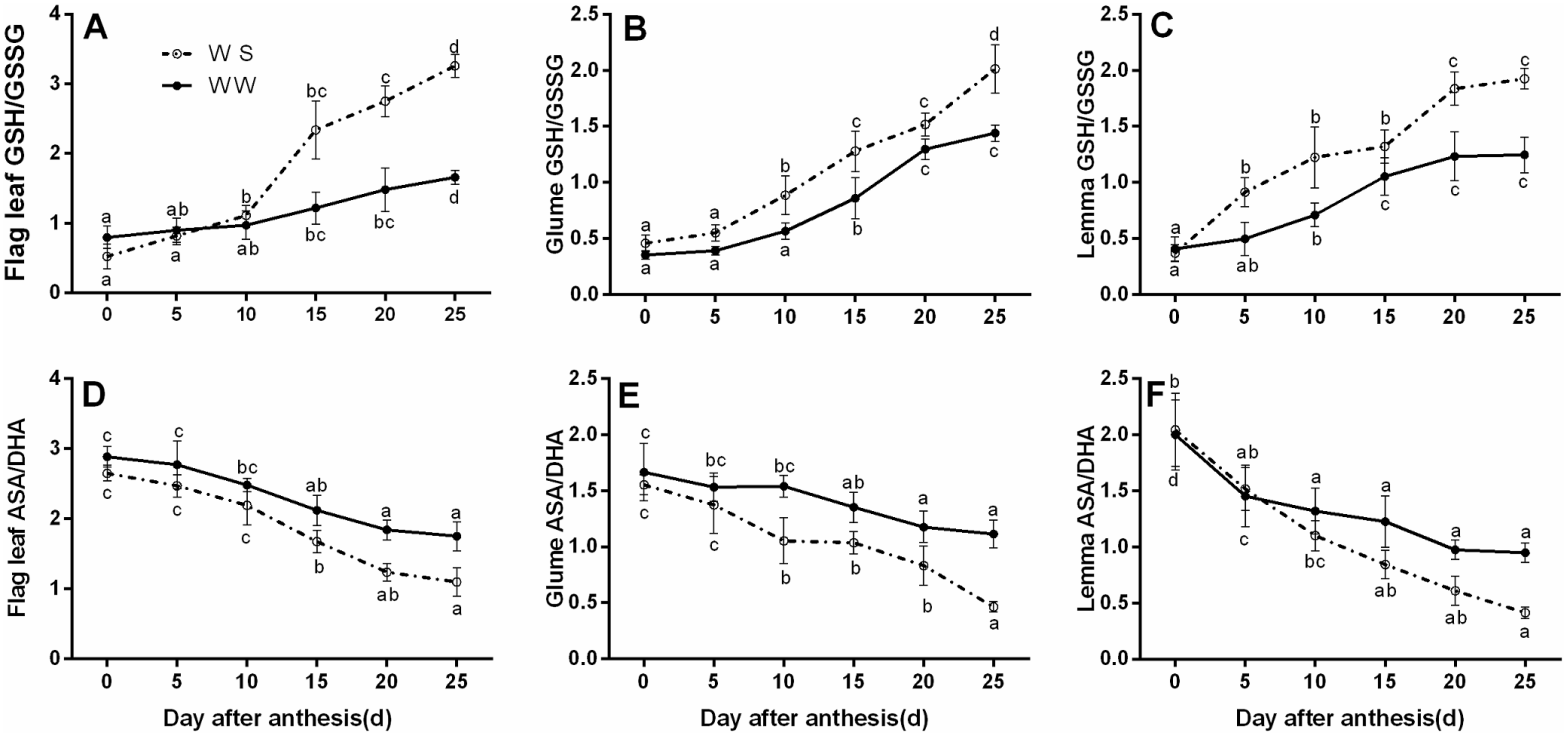
Effects of drought stress on the ratios of GSH/GSSG (A, B, and C) and ASA/DHA (D, E, and F) of the flag leaves, glumes, and lemmas at the grain-filling stage under well-watered (WW) and water-stressed (WS) conditions. All data represent means ± standard deviations (SD) of three replicates. Values with different letters indicated the significant differences at *P < 0*.*05* level in each stress treatment through time according to Duncan’s multiple range test.

The GSH/GSSG ratio in the flag leaves and spike bracts gradually increased during the early and middle grain filling stages, reaching a maximum at the late grain-filling stage and then maintained or slightly increased in WW condition, and this ratio increased under WS condition **(**Fig 3A–3C**)**. Under water deficit, the ratio of GSH/GSSG in the flag leaves rose by 64.2% on DAA 25 compared with the WW treated condition, whereas in glumes and lemmas it rose by 39.6% and 54.8% respectively (Fig 3D–3F). In contrast to these results, the ASA/DHA ratio of the flag leaves and spike bracts decreased gradually during the grain-filling stage, and under WW condition, the ratio of ASA/DHA in the flag leaves decreased by 51.6% on DAA 20, whereas in glumes and lemmas it decreased by 43.2% and 35.8% respectively. It was found that the GSH/GSSG ratio and ASA/DHA ratio were maintained more effectively in glumes and lemmas compared with the flag leaves. These results suggested that the role of spike organs can not be ignored in balancing the redox state of ascorbate and glutathione under water stress.

### Activities of antioxidant enzymes in the ASA-GSH cycle

**Fig 4:**
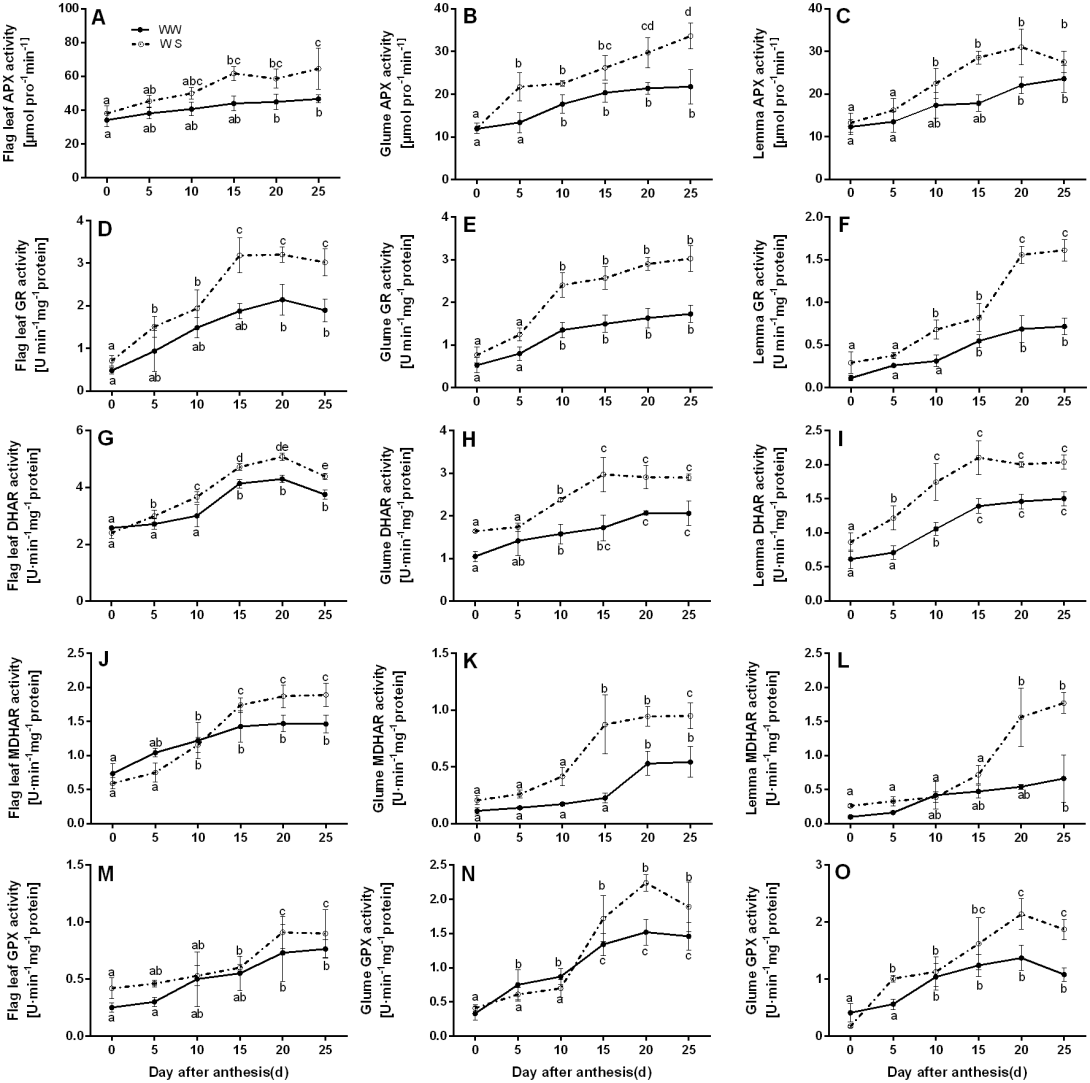
Effect of water deficit on antioxidant enzymes (APX, GR, DHAR, MDHAR, and GPX) activities involved in ASA-GSH metabolism in the flag leaves, glumes, and lemmas of wheat at the grain-filling stage under well-watered (WW) and water-stressed (WS) conditions. All data represent means ± standard deviations (SD) of three replicates. Values with different letters indicated the significant differences at *P < 0*.*05* level in each stress treatment through time according to Duncan’s multiple range test.

To better understand the roles of antioxidant enzymes in wheat grain filling, the activities of five enzymes involved in the ASA-GSH cycle in the flag leaves and spike bracts were determined. Fig 4 showed that drought enhanced the activities of APX, GR, DHAR, MDHAR, and GPX in all three organs, compared with WW-treated. The activities of APX, GR, DHAR, and MDHAR, remained relatively stable throughout the experimental period (except for DHAR activity in the flag leaves and MDHAR activity in glumes) in WW-treated wheat. While the drought stress had a strengthening effect on the five antioxidant enzyme activities and varies in different organs. Beside the promotion of enzyme activities under WS condition, the spike bracts had more enhancement of enzymes activities than flag leaves. For instance, the GPX activities in the flag leaves transiently increased during middle and late grain filling periods, reaching a maximum at 20 DAA, and decreasing thereafter. The glumes and lemmas showed similar trends. Under water deficit, the activity of GPX in the flag leaves rose by 24.7% on 25 DAA compared with the WW treated condition, whereas it rose by 47.4% and 56.2% respectively in glumes and lemmas (Fig 4M–4O). Thus, we can conclude that water deficit promotes the activities of ASA-GSH cycle enzymes and promotes more in spike bracts, which control ROS content and cellular redox homeostasis better.

### The expression of *APX1*, *GRC1*, *DHAR*, *MDHAR*, *GPX1*, and *GS3* in ASA-GSH cycle

**Fig 5:**
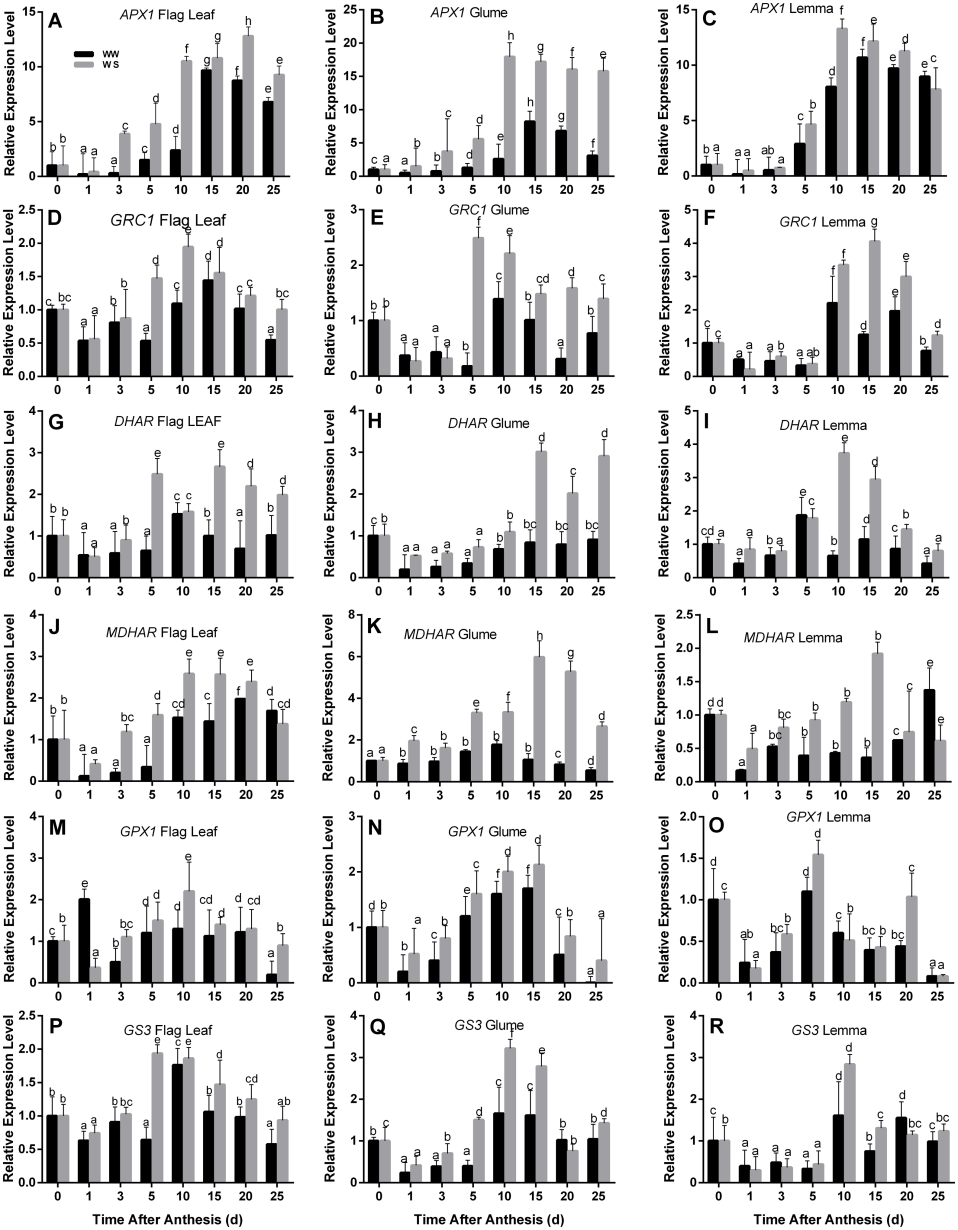
Effects of drought on transcript levels of six genes encoding ASA-GSH cycle enzymes in the flag leaves, glumes, and lemmas of wheat at the grain-filling stage under well-watered (WW) and water-stressed (WS) conditions. Transcripts were analyzed by qRT-PCR using *Tubulin* gene as internal control. Each value is the mean ± standard deviation of three independent measurements. Values with different letters indicated the significant differences at *P < 0*.*05* level in each stress treatment through time according to Duncan’s multiple range test.

Comparative transcript analysis was performed in the flag leaves, glumes and lemmas to analyse changes in ASA-GSH pathways under water deficit. The *APX1*, *GRC1*, *DHAR*, *MDHAR*, *GPX1*, and *GS3* transcript levels were measured using RT-PCR with the *Tubulin* gene as the internal control (Fig 5). The *APX1* expression in all three organs was steadily increased from 1 to 15 DAA, and then slowly decreased in WW condition. While in WS condition, the *APX1* expression in the flag leaves peaked at 20 DAA and in glumes and lemmas at 10 DAA (Fig 5A–5C). In glumes, the *GRC1* expression was steeply up-regulated at 5 DAA (10.7 fold) and slightly up-regulated thereafter under WS condition compared to WW condition, and the extent of induced was higher than that of the flag leaves (2.77 fold) and lemmas (1.15 fold) (Fig 5D–5F). The WS treatment increased the *DHAR* expression in the flag leaves by 166% and in glumes by 262% compared to the WW treated wheat at 15 DAA(Fig 5G and 5H). The *MDHAR* expression at 15 DAA increased in the flag leaves (0.79 fold), glumes (4.69 fold) and lemmas (4.33 fold) under WS condition compared to the WW, respectively (Fig 5J–5L). *GPX1* and *GS3* genes exhibited similar expression patterns in all three organs of wheat under both water conditions. Transcript levels of these six genes were enhanced in drought stress, peaked at 10 or 15 DAA, and slowly or rapidly decreased thereafter (except for *GRC1* expression level in glumes). Beside the up-regulated expression levels under water deficit, the spike bracts displayed generally higher transcript levels compared with the flag leaves. This were entirely consistent with the results that ASA-GSH cycle related enzymes activities were strongly increased in glumes and lemmas at the late phase of grain filling.

## Discussion

Winter wheat is the second largest widely cultivated crops in China, where drought is the major abiotic stress limiting the yield of wheat [9, 24]. Water deficit occurred during the grain-filling phase, which has an adverse impact on wheat productivity and quality [2, 8, 10]. Wheat cultivars have adapted various drought tolerance mechanisms, which include formation of deeper roots, accumulation of higher biomass, exertion of better stomatal control over transpiration, enhancement of osmoprotective and antioxidant response [65], and importantly a better coordination of positive and negative regulation of gene expression [66]. It has been suggested that the ear photosynthesis of wheat can provide ranging from 20 to 40% of the total grain carbon, depending on genotype and growing conditions, for the photosynthesis in spike green parts (e.g. bracts, awns) were important sources of assimilate [14, 19, 67]. However, spike photosynthesis involved in drought tolerance was largely unknown.

### Changes of photosynthetic parameters under drought stress

Recent findings highlight the significance of ear photosynthesis in final grain yields and ear photosynthesis shows higher drought tolerance compared to the flag leaves [30, 68]. In present study, we found that the *P*_N_, *E*, and *g*_s_ of the flag leaves were sharply decreased by exposure to drought conditions; whereas in the ear, these processes did not show obvious decline until after 10 DAA (Fig 1). It suggested that spike photosynthesis was less sensitive to drought stress compared with the flag leaves, contributing largely to the grain yields. In order to maintain a high and stable photosynthetic rate, the *C*_i_ showed an increasing trend during the grain-filling period in the flag leaves and spikes. It was accordance with the findings in potato [69], muskmelon [70] and wheat [71]. Spike photosynthesis was transported assimilates to grain. The *Ci* decreased at 25 DAA in spikes because of the end of grain filling. It is worth noting that the potential contribution from awn photosynthesis for the increase of photosynthetic surface area [30, 72]. Post-anthesis water stress substantially accelerated the chlorophyll destroy and the decrease of RWC in the flag leaves, but the reduction of these parameters was much less influenced by drought in ear bracts (Fig 2). Stomatal control is important for regulation of both water loss and CO_2_ assimilation in response to drought stress. Here, the higher stomatal conductance was in accordance with the higher rates of photosynthesis [73]. A reduction in leaf water potential will reduce stomatal conductance and eventually inhibit photosynthetic metabolism [74]. This indicates that retention of photosynthetic components in spikes under drought conditions might help ear to continue as a source of assimilates at the late grain-filling stage, when the flag leaves photosynthetic performance become negligible. Alternatively, drought may accelerate senescence in the flag leaves due to inhibit the synthesis of chlorophyll and breakdown of thylakoid components, for senescence normally start at the older leaves at the top of the stem [75]. Given the spike bracts are the latest photosynthetic organs to develop in wheat, it is reasonable to propose that the glumes and lemmas senesced later than the flag leaves of wheat Pubing143 at the late grain-filling stage.

### Enzymes activities involved in ASA-GSH metabolism

Drought stress inevitably raised oxidative stress, resulting in enhanced ROS accumulation, especially H_2_O_2_ in chloroplasts. Respond to oxidative stress, plants evolved complex acclimation and defence strategies to minimize the deleterious effects due to excess ROS [41]. It has been proposed that ASA, GSH, and the enzymes including APX, GR, DHAR, MDHAR, and GPX involved in ASA-GSH metabolism enable to act as ROS scavengers [76, 77], and the involvement of the ASA-GSH cycle in the protection of oxidative damage caused by drought to plants has also been observed in *Fargesia rufa* [78], soybean [79], *Cerasus humilis* [33]. Unfortunately, none of these studies tried to elucidate ROS defence and redox regulation mechanisms in wheat spike bracts under drought conditions in detail. We compared the changes of enzymes involved in the ASA-GSH cycle in the flag leaves and ear bracts during grain filling under water stress conditions. We observed that the spike organs were capable of scavenging excessive ROS, and keeping lower MDA and H_2_O_2_ contents under WS condition (Table 2). This might be due to the higher increased percentages of antioxidant enzymes activities such as APX, GR, DHAR, MDHAR and GPX involved in ASA-GSH cycle in spike bracts than in the flag leaves under WS condition (Fig 4). Besides, we also found that drought stress increased the GSH/GSSG ratio and reduced the ASA/DHA ratio. The extent of increase and decrease was much higher in ear bracts than that in the flag leaves (Fig 3). Thus, the function of ASA-GSH cycle could not be ignored in alleviating oxidative damage by scavenging more excess ROS in spikes under drought stress. This could also be the reasonable explanation for the more stable photosynthetic capacity in spikes, compared with the flag leaves. It was in accordance with the findings of Kong [31] and Kohl [80].

### Gene relative expression involved in ASA-GSH cycle under drought stress

Under water stress conditions, not only ROS in plants increases rapidly, but also the gene expression of antioxidant enzymes were induced to cope with the stress. Few previous studies focused on related genes of the ASA-GSH cycle in wheat seeding [46–48], but comprehensive studies on spikes have not been presented so far in winter wheat. In our study, we investigated the expression of genes related to ASA-GSH cycle (*APX1*, *GRC1*, *DHAR*, *MDHAR*, *GPX1*, and *GS3*) in the flag leaves and ear bracts of wheat under different water conditions to evaluate its role in resisting oxidative damage during grain filling (Fig 5). Transcript levels of six genes were up-regulated by drought in both flag leaves and spike bracts, but a greater increase was detected in spike bracts. The results indicate that more transcription of ASA-GSH based detoxification machinery in ear bracts may serve to adapt the abiotic stresses like limited water supply and avoid the adverse shift of the cellular redox balance. The spike bracts are considered to have the capacity of stay-green and mediating the ROS accumulation [24], but no experimental evidence is available to support this hypothesis. In current study, drought stress may have helped to increase the transcript levels of the genes involved in ASA-GSH cycle, which were highly expressed in spike bracts at the late grain-filling stage. However, further studies are needed to elucidate the mechanism of transcriptional regulation of genes involved in ASA-GSH metabolism in ears. Perhaps this is one of the reasons that ear has attributed that confer resistance to water stress suggested by Wang [19], Farooq [2], Merah and Monneveux [18] and Hein [30]. According to these authors, the spike is the main source of assimilates during grain filling under drought stress.

In conclusion, the wheat spikes exhibited a more stable photosynthetic capability through the water regulation ability, and also presented the competence as important as flag leaves in response to drought stress by the ASA-GSH cycle during the grain-filling stage. Our findings suggested that spikes have a considerable role in response to water deficit through the ASA-GSH cycle. It provided the possible strategy to improve wheat drought tolerance by genetic engineering in the future.

## Supporting information

S1 Fig The morphology of wheat ear. (TIF)
