# Supplementary figures and images for: Photosynthetic and ascorbate-glutathione metabolism in the flag leaves as compared to spikes under drought stress of winter wheat (Triticum aestivum L.)
Source: PLoS One. 2018 Mar 22;13(3):e0194625. doi: 10.1371/journal.pone.0194625 (PMC5864061; doi:10.1371/journal.pone.0194625)

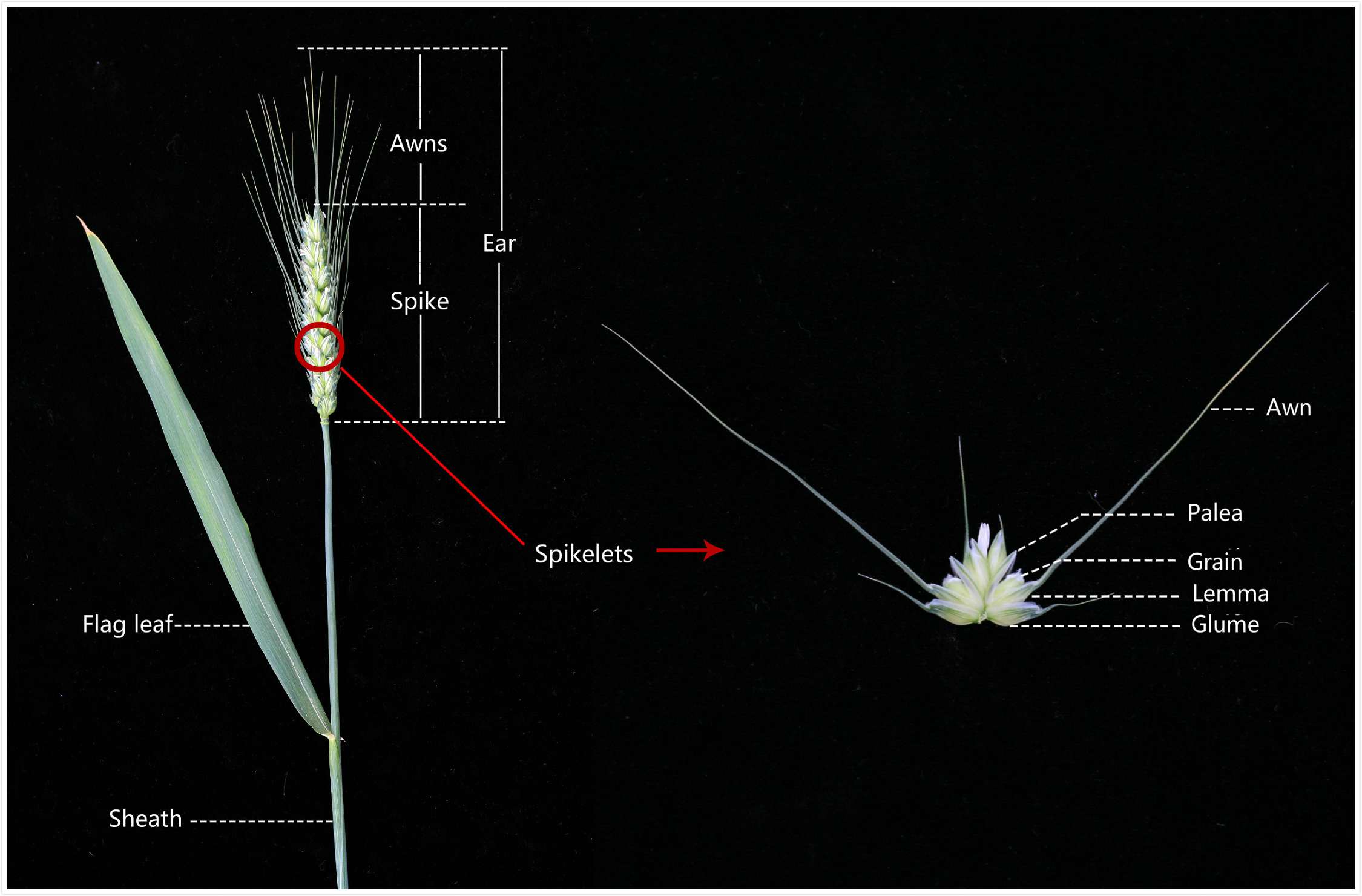

Supplement: S1 Fig — (TIF) [file pone.0194625.s001.tif]
